# [(*Z*)-*O*-Ethyl *N*-(4-nitro­phen­yl)thio­carbamato-κ*S*](triphenyl­phosphine-κ*P*)gold(I) dichloro­methane solvate

**DOI:** 10.1107/S1600536809043876

**Published:** 2009-10-31

**Authors:** Soo Yei Ho, Edward R. T. Tiekink

**Affiliations:** a Department of Chemistry, National University of Singapore, Singapore 117543; b Department of Chemistry, University of Malaya, 50603 Kuala Lumpur, Malaysia

## Abstract

An *S*,*P*-donor set in the title solvate, [Au(C_9_H_9_N_2_O_3_S)(C_18_H_15_P)]·CH_2_Cl_2_, defines a linear geometry for the Au^I^ atom [S—Au—P = 177.75 (7)°], with the minor distortion ascribed to the influence of an intra­molecular Au⋯O contact [3.019 (6) Å]. In the crystal, the packing is stabilized by a network of C—H⋯S, C—H⋯N and C—H⋯O contacts.

## Related literature

For structural systematics and luminescence properties of phosphinegold(I) carbonimidothio­ates, see: Ho *et al.* (2006 ▶); Ho & Tiekink (2007 ▶); Kuan *et al.* (2008 ▶). For the synthesis, see: Hall *et al.* (1993 ▶).
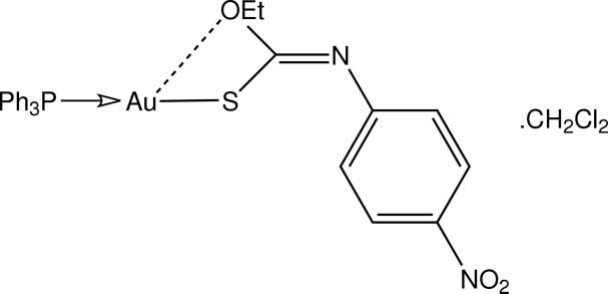


## Experimental

### 

#### Crystal data

[Au(C_9_H_9_N_2_O_3_S)(C_18_H_15_P)]·CH_2_Cl_2_
*M* *_r_* = 769.40
Triclinic, 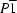

*a* = 8.7525 (7) Å
*b* = 11.1373 (9) Å
*c* = 15.8981 (13) Å
α = 104.311 (2)°
β = 105.559 (2)°
γ = 91.775 (2)°
*V* = 1438.7 (2) Å^3^
*Z* = 2
Mo *K*α radiation
μ = 5.46 mm^−1^
*T* = 238 K
0.39 × 0.34 × 0.10 mm

#### Data collection

Bruker SMART CCD diffractometer
Absorption correction: multi-scan (*SADABS*; Bruker, 2000 ▶) *T*_min_ = 0.561, *T*_max_ = 1
10007 measured reflections
6534 independent reflections
5162 reflections with *I* > 2σ(*I*)
*R*_int_ = 0.064

#### Refinement

*R*[*F* ^2^ > 2σ(*F* ^2^)] = 0.055
*wR*(*F* ^2^) = 0.128
*S* = 0.94
6534 reflections
343 parameters
H-atom parameters constrained
Δρ_max_ = 2.69 e Å^−3^
Δρ_min_ = −1.19 e Å^−3^

### 

Data collection: *SMART* (Bruker, 2000 ▶); cell refinement: *SAINT* (Bruker, 2000 ▶); data reduction: *SAINT*; program(s) used to solve structure: *PATTY* in *DIRDIF92* (Beurskens *et al.*, 1992 ▶); program(s) used to refine structure: *SHELXL97* (Sheldrick, 2008 ▶); molecular graphics: *ORTEP-3* (Farrugia, 1997 ▶) and *DIAMOND* (Brandenburg, 2006 ▶); software used to prepare material for publication: *SHELXL97*.

## Supplementary Material

Crystal structure: contains datablocks global, I. DOI: 10.1107/S1600536809043876/hb5169sup1.cif

Structure factors: contains datablocks I. DOI: 10.1107/S1600536809043876/hb5169Isup2.hkl

Additional supplementary materials:  crystallographic information; 3D view; checkCIF report

## Figures and Tables

**Table 1:** Selected bond lengths (Å)

Au—S1	2.3019 (19)
Au—P1	2.2545 (18)

**Table 2:** Hydrogen-bond geometry (Å, °)

*D*—H⋯*A*	*D*—H	H⋯*A*	*D*⋯*A*	*D*—H⋯*A*
C23—H23⋯N1^i^	0.94	2.55	3.318 (11)	139
C14—H14⋯O3^ii^	0.94	2.47	3.366 (12)	160
C28—H28a⋯O1^iii^	0.98	2.52	3.330 (13)	140
C28—H28b⋯S1^iv^	0.98	2.86	3.617 (11)	134

Symmetry codes: (i) 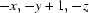; (ii) 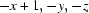; (iii) 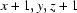; (iv) 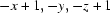.

**Fig. 1.**
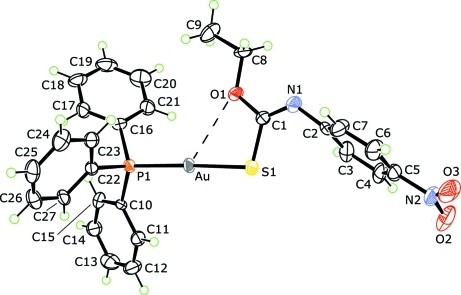
Molecular structure of (I) showing displacement ellipsoids at the 50% probability level.

**Fig. 2.**
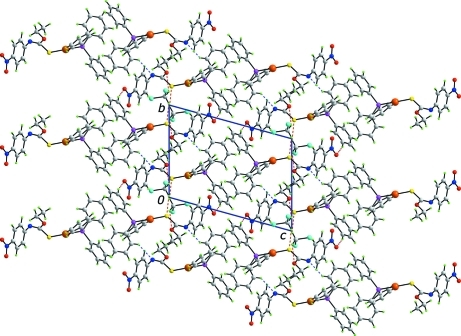
Unit-cell contents for (I) viewed in projection down the a axis. Colour code: Au, orange; Cl, cyan; S, yellow; P, pink; O, red; N, blue; C, grey; and H, green.

## supplementary crystallographic information

### Comment

As a continuation of studies into the structural systematics of molecules with the general formula *R*_3_PAu[SC(OR')NR''] for *R*, *R*' and *R*'' = alkyl and aryl (Ho *et al.* 2006; Ho & Tiekink, 2007; Kuan *et al.*, 2008), the title dichloromethane solvate, (I), was characterized. The Au atom in (I) exists in the expected linear geometry defined by S and P atoms, Table 1 and Fig. 1, with the deviation from the ideal 180° angle being related to the close approach of the O1 atom, 3.019 (6) Å. The structure follows closely literature precedents.

The crystal structure of (I) is stabilized by a series of large rings mediated by C—H···S, O and N contacts, Table 2 and Fig. 2. Thus, C_phenyl_—*H*···O_nitro_ contacts link centrosymmetrically related molecules *via* 30-membered {···ONC_4_NCSAuPC_3_H}_2_ synthons. Smaller centrosymmetric rings are formed through the agency of C_phenyl_—*H*···N_imine_ contacts that lead to 16-membered {···NCSAuPC_2_H}_2_ synthons. Centrosymmetrically related dichloromethane molecules bridge a pair of complex molecules, forming C—H···O1, S1 contacts, leading to the formation of 12-membered {···OCS···HCH}_2_ synthons.

### Experimental

The unsolvated compound was prepared following the standard literature procedure from the reaction of Ph_3_PAuCl and EtOC(S)N(H)C_6_H_4_NO_2_-4 in the presence of base (Hall *et al.*, 1993); m. pt. 423–425 K. Analysis for C_27_H_24_AuN_2_O_3_PS: found (calculated): C: 47.25 (47.38); H: 3.39 (3.53); N: 4.33 (4.09); S: 4.50 (4.68). IR (cm^-1^): ν(C—S) 1102 s, 849*m*; ν(C—N) 1582 s; ν(C—O) 1145*m*. ^31^P{^1^H} NMR: δ 37.7 p.p.m. Yellow crystals of the dichloromethane solvate (I) were obtained from the layering of ethanol on a dichloromethane solution of the characterized product.

### Refinement

The H atoms were geometrically placed (C—H = 0.94–0.98 Å) and refined as riding with *U*_iso_(H) = 1.2–1.5*U*_eq_(C). The maximum and minimum residual electron density peaks of 2.69 and 1.19 e Å^-3^, respectively, were located 0.99 Å and 0.97 Å from the Au atom.

### Crystal data

**Table:**

[Au(C_9_H_9_N_2_O_3_S)(C_18_H_15_P)]·CH_2_Cl_2_	*Z* = 2
*M**_r_* = 769.40	*F*(000) = 752
Triclinic, *P*1	*D*_x_ = 1.776 Mg m^−^^3^
Hall symbol: -P 1	Mo *K*α radiation, λ = 0.71069 Å
*a* = 8.7525 (7) Å	Cell parameters from 3380 reflections
*b* = 11.1373 (9) Å	θ = 2.7–25.5°
*c* = 15.8981 (13) Å	µ = 5.46 mm^−^^1^
α = 104.311 (2)°	*T* = 238 K
β = 105.559 (2)°	Block, yellow
γ = 91.775 (2)°	0.39 × 0.34 × 0.10 mm
*V* = 1438.7 (2) Å^3^	

### Data collection

**Table:**

Bruker SMART CCD diffractometer	6534 independent reflections
Radiation source: fine-focus sealed tube	5162 reflections with *I* > 2σ(*I*)
graphite	*R*_int_ = 0.064
ω scans	θ_max_ = 27.5°, θ_min_ = 1.4°
Absorption correction: multi-scan (*SADABS*; Bruker, 2000)	*h* = −11→11
*T*_min_ = 0.561, *T*_max_ = 1	*k* = −12→14
10007 measured reflections	*l* = −20→18

### Refinement

**Table:**

Refinement on *F*^2^	Primary atom site location: structure-invariant direct methods
Least-squares matrix: full	Secondary atom site location: difference Fourier map
*R*[*F*^2^ > 2σ(*F*^2^)] = 0.055	Hydrogen site location: inferred from neighbouring sites
*wR*(*F*^2^) = 0.128	H-atom parameters constrained
*S* = 0.94	*w* = 1/[σ^2^(*F*_o_^2^) + (0.0474*P*)^2^] where *P* = (*F*_o_^2^ + 2*F*_c_^2^)/3
6534 reflections	(Δ/σ)_max_ < 0.001
343 parameters	Δρ_max_ = 2.69 e Å^−^^3^
0 restraints	Δρ_min_ = −1.19 e Å^−^^3^

### Special details

**Table:**

Geometry. All s.u.'s (except the s.u. in the dihedral angle between two l.s. planes) are estimated using the full covariance matrix. The cell s.u.'s are taken into account individually in the estimation of s.u.'s in distances, angles and torsion angles; correlations between s.u.'s in cell parameters are only used when they are defined by crystal symmetry. An approximate (isotropic) treatment of cell s.u.'s is used for estimating s.u.'s involving l.s. planes.
Refinement. Refinement of *F*^2^ against ALL reflections. The weighted *R*-factor *wR* and goodness of fit *S* are based on *F*^2^, conventional *R*-factors *R* are based on *F*, with *F* set to zero for negative *F*^2^. The threshold expression of *F*^2^ > 2σ(*F*^2^) is used only for calculating *R*-factors(gt) *etc*. and is not relevant to the choice of reflections for refinement. *R*-factors based on *F*^2^ are statistically about twice as large as those based on *F*, and *R*- factors based on ALL data will be even larger.

### Fractional atomic coordinates and isotropic or equivalent isotropic displacement parameters (Å^2^)

**Table:**

	*x*	*y*	*z*	*U*_iso_*/*U*_eq_	
Au	0.08336 (3)	0.29401 (3)	0.155405 (19)	0.02476 (11)	
S1	0.1369 (2)	0.2101 (2)	0.02002 (14)	0.0337 (5)	
P1	0.0409 (2)	0.37531 (18)	0.29087 (13)	0.0227 (4)	
O1	−0.0966 (6)	0.3429 (5)	−0.0214 (4)	0.0304 (12)	
O2	0.3929 (8)	−0.1228 (7)	−0.3297 (5)	0.061 (2)	
O3	0.4680 (9)	0.0425 (7)	−0.3643 (5)	0.066 (2)	
N1	0.0125 (8)	0.2734 (6)	−0.1360 (5)	0.0347 (16)	
N2	0.3922 (9)	−0.0126 (8)	−0.3252 (5)	0.048 (2)	
C1	0.0107 (9)	0.2789 (7)	−0.0565 (5)	0.0289 (17)	
C2	0.1120 (9)	0.2009 (7)	−0.1789 (5)	0.0281 (17)	
C3	0.1034 (10)	0.0715 (8)	−0.1934 (6)	0.038 (2)	
H3	0.0336	0.0315	−0.1707	0.046*	
C4	0.1951 (9)	0.0029 (8)	−0.2401 (6)	0.037 (2)	
H4	0.1889	−0.0840	−0.2493	0.044*	
C5	0.2968 (9)	0.0618 (8)	−0.2736 (6)	0.0337 (19)	
C6	0.3108 (11)	0.1898 (9)	−0.2601 (6)	0.044 (2)	
H6	0.3810	0.2292	−0.2830	0.053*	
C7	0.2176 (11)	0.2575 (8)	−0.2117 (6)	0.038 (2)	
H7	0.2264	0.3446	−0.2008	0.046*	
C8	−0.2083 (10)	0.4005 (8)	−0.0801 (6)	0.035 (2)	
H8A	−0.2696	0.3377	−0.1349	0.042*	
H8B	−0.1517	0.4629	−0.0979	0.042*	
C9	−0.3173 (11)	0.4610 (9)	−0.0275 (7)	0.047 (2)	
H9A	−0.3948	0.5008	−0.0648	0.070*	
H9B	−0.2552	0.5230	0.0264	0.070*	
H9C	−0.3722	0.3983	−0.0101	0.070*	
C10	0.0794 (8)	0.2722 (6)	0.3657 (5)	0.0175 (14)	
C11	−0.0100 (9)	0.2683 (7)	0.4250 (5)	0.0289 (17)	
H11	−0.0928	0.3197	0.4274	0.035*	
C12	0.0222 (10)	0.1895 (8)	0.4802 (6)	0.0348 (19)	
H12	−0.0391	0.1861	0.5200	0.042*	
C13	0.1461 (11)	0.1148 (8)	0.4770 (6)	0.043 (2)	
H13	0.1698	0.0627	0.5162	0.051*	
C14	0.2344 (10)	0.1157 (8)	0.4174 (6)	0.040 (2)	
H14	0.3160	0.0633	0.4144	0.048*	
C15	0.2008 (9)	0.1957 (8)	0.3617 (6)	0.0328 (19)	
H15	0.2607	0.1979	0.3210	0.039*	
C16	−0.1630 (8)	0.4119 (7)	0.2803 (5)	0.0244 (16)	
C17	−0.1997 (10)	0.5095 (8)	0.3435 (5)	0.0346 (19)	
H17	−0.1180	0.5601	0.3917	0.042*	
C18	−0.4767 (11)	0.4562 (10)	0.2640 (7)	0.049 (3)	
H18	−0.5836	0.4713	0.2583	0.059*	
C19	−0.3572 (11)	0.5311 (9)	0.3346 (6)	0.043 (2)	
H19	−0.3830	0.5969	0.3767	0.052*	
C20	−0.4417 (10)	0.3609 (10)	0.2025 (8)	0.054 (3)	
H20	−0.5240	0.3094	0.1553	0.065*	
C21	−0.2840 (9)	0.3400 (8)	0.2099 (6)	0.037 (2)	
H21	−0.2596	0.2759	0.1661	0.045*	
C22	0.1646 (8)	0.5220 (7)	0.3530 (5)	0.0241 (16)	
C23	0.1571 (9)	0.6150 (7)	0.3100 (5)	0.0281 (17)	
H23	0.0886	0.6027	0.2512	0.034*	
C24	0.2522 (11)	0.7291 (8)	0.3543 (6)	0.042 (2)	
H24	0.2473	0.7942	0.3260	0.051*	
C25	0.3540 (11)	0.7435 (8)	0.4411 (6)	0.041 (2)	
H25	0.4198	0.8186	0.4710	0.049*	
C26	0.3595 (9)	0.6500 (8)	0.4834 (6)	0.0351 (19)	
H26	0.4270	0.6619	0.5424	0.042*	
C27	0.2666 (9)	0.5388 (8)	0.4397 (5)	0.0293 (17)	
H27	0.2719	0.4740	0.4685	0.035*	
C28	0.6962 (11)	0.0796 (10)	0.9653 (7)	0.052 (3)	
H28A	0.7867	0.1437	0.9962	0.063*	
H28B	0.7087	0.0138	0.9970	0.063*	
Cl1	0.5199 (3)	0.1453 (2)	0.9715 (2)	0.0559 (7)	
Cl2	0.6994 (4)	0.0171 (4)	0.8542 (2)	0.0823 (10)	

### Atomic displacement parameters (Å^2^)

**Table:**

	*U*^11^	*U*^22^	*U*^33^	*U*^12^	*U*^13^	*U*^23^
Au	0.02296 (16)	0.03345 (18)	0.01681 (16)	0.00492 (11)	0.00790 (11)	0.00206 (11)
S1	0.0337 (11)	0.0484 (12)	0.0218 (10)	0.0176 (10)	0.0134 (9)	0.0068 (9)
P1	0.0173 (9)	0.0305 (10)	0.0186 (10)	0.0038 (8)	0.0062 (7)	0.0019 (8)
O1	0.029 (3)	0.038 (3)	0.029 (3)	0.015 (2)	0.014 (2)	0.010 (2)
O2	0.057 (5)	0.050 (4)	0.070 (5)	0.012 (4)	0.034 (4)	−0.011 (4)
O3	0.071 (5)	0.092 (6)	0.065 (5)	0.044 (5)	0.051 (4)	0.036 (4)
N1	0.039 (4)	0.040 (4)	0.031 (4)	0.016 (3)	0.018 (3)	0.011 (3)
N2	0.034 (4)	0.065 (6)	0.036 (5)	0.020 (4)	0.012 (4)	−0.003 (4)
C1	0.024 (4)	0.038 (5)	0.026 (4)	0.013 (3)	0.010 (3)	0.007 (3)
C2	0.031 (4)	0.039 (5)	0.011 (4)	0.015 (4)	0.002 (3)	0.004 (3)
C3	0.035 (5)	0.042 (5)	0.032 (5)	−0.004 (4)	0.013 (4)	−0.004 (4)
C4	0.031 (4)	0.033 (4)	0.034 (5)	−0.002 (4)	0.008 (4)	−0.014 (4)
C5	0.022 (4)	0.049 (5)	0.027 (4)	0.008 (4)	0.008 (3)	0.003 (4)
C6	0.045 (5)	0.057 (6)	0.044 (6)	0.017 (5)	0.028 (5)	0.021 (5)
C7	0.051 (5)	0.038 (5)	0.042 (5)	0.022 (4)	0.028 (5)	0.022 (4)
C8	0.034 (4)	0.044 (5)	0.034 (5)	0.025 (4)	0.012 (4)	0.018 (4)
C9	0.045 (5)	0.047 (5)	0.062 (7)	0.021 (5)	0.036 (5)	0.014 (5)
C10	0.016 (3)	0.019 (3)	0.014 (3)	0.003 (3)	−0.001 (3)	0.004 (3)
C11	0.031 (4)	0.027 (4)	0.030 (4)	0.003 (3)	0.014 (4)	0.001 (3)
C12	0.037 (5)	0.042 (5)	0.030 (5)	0.007 (4)	0.014 (4)	0.012 (4)
C13	0.050 (5)	0.037 (5)	0.044 (6)	0.003 (4)	0.005 (5)	0.024 (4)
C14	0.038 (5)	0.034 (5)	0.049 (6)	0.014 (4)	0.010 (4)	0.012 (4)
C15	0.022 (4)	0.048 (5)	0.027 (4)	0.010 (4)	0.010 (3)	0.003 (4)
C16	0.022 (4)	0.024 (4)	0.028 (4)	0.006 (3)	0.006 (3)	0.010 (3)
C17	0.031 (4)	0.049 (5)	0.023 (4)	0.012 (4)	0.011 (4)	0.004 (4)
C18	0.029 (5)	0.073 (7)	0.055 (7)	0.023 (5)	0.011 (5)	0.031 (6)
C19	0.040 (5)	0.058 (6)	0.044 (6)	0.031 (5)	0.023 (5)	0.022 (5)
C20	0.017 (4)	0.073 (7)	0.064 (7)	0.004 (4)	0.003 (4)	0.011 (6)
C21	0.024 (4)	0.047 (5)	0.031 (5)	0.007 (4)	0.002 (4)	−0.001 (4)
C22	0.019 (3)	0.030 (4)	0.020 (4)	0.004 (3)	0.006 (3)	0.001 (3)
C23	0.030 (4)	0.031 (4)	0.026 (4)	0.005 (3)	0.012 (3)	0.007 (3)
C24	0.052 (6)	0.039 (5)	0.045 (6)	0.006 (4)	0.030 (5)	0.010 (4)
C25	0.042 (5)	0.032 (5)	0.039 (5)	−0.011 (4)	0.012 (4)	−0.008 (4)
C26	0.024 (4)	0.046 (5)	0.028 (5)	−0.009 (4)	0.006 (3)	0.002 (4)
C27	0.030 (4)	0.040 (5)	0.016 (4)	0.006 (4)	0.007 (3)	0.003 (3)
C28	0.031 (5)	0.058 (6)	0.065 (7)	0.006 (5)	0.009 (5)	0.015 (5)
Cl1	0.0490 (14)	0.0489 (14)	0.0709 (19)	0.0094 (12)	0.0188 (13)	0.0154 (13)
Cl2	0.0596 (18)	0.116 (3)	0.064 (2)	0.0261 (18)	0.0179 (16)	0.0083 (18)

### Geometric parameters (Å, °)

**Table:**

Au—S1	2.3019 (19)	C12—C13	1.391 (12)
Au—P1	2.2545 (18)	C12—H12	0.9400
S1—C1	1.755 (8)	C13—C14	1.376 (13)
P1—C16	1.816 (7)	C13—H13	0.9400
P1—C10	1.825 (7)	C14—C15	1.389 (12)
P1—C22	1.826 (8)	C14—H14	0.9400
O1—C1	1.352 (8)	C15—H15	0.9400
O1—C8	1.444 (9)	C16—C21	1.373 (11)
O2—N2	1.212 (10)	C16—C17	1.396 (10)
O3—N2	1.260 (11)	C17—C19	1.383 (11)
N1—C1	1.255 (10)	C17—H17	0.9400
N1—C2	1.401 (9)	C18—C20	1.356 (13)
N2—C5	1.460 (10)	C18—C19	1.380 (13)
C2—C7	1.382 (11)	C18—H18	0.9400
C2—C3	1.397 (12)	C19—H19	0.9400
C3—C4	1.362 (11)	C20—C21	1.386 (11)
C3—H3	0.9400	C20—H20	0.9400
C4—C5	1.377 (12)	C21—H21	0.9400
C4—H4	0.9400	C22—C23	1.370 (11)
C5—C6	1.385 (12)	C22—C27	1.390 (10)
C6—C7	1.383 (11)	C23—C24	1.408 (12)
C6—H6	0.9400	C23—H23	0.9400
C7—H7	0.9400	C24—C25	1.395 (13)
C8—C9	1.500 (10)	C24—H24	0.9400
C8—H8A	0.9800	C25—C26	1.367 (13)
C8—H8B	0.9800	C25—H25	0.9400
C9—H9A	0.9700	C26—C27	1.374 (11)
C9—H9B	0.9700	C26—H26	0.9400
C9—H9C	0.9700	C27—H27	0.9400
C10—C11	1.385 (10)	C28—Cl2	1.740 (11)
C10—C15	1.387 (9)	C28—Cl1	1.743 (9)
C11—C12	1.373 (11)	C28—H28A	0.9800
C11—H11	0.9400	C28—H28B	0.9800
			
P1—Au—S1	177.75 (7)	C11—C12—H12	120.1
C1—S1—Au	104.1 (2)	C13—C12—H12	120.1
C16—P1—C10	106.1 (3)	C14—C13—C12	121.1 (8)
C16—P1—C22	105.1 (3)	C14—C13—H13	119.5
C10—P1—C22	106.5 (3)	C12—C13—H13	119.5
C16—P1—Au	112.4 (3)	C13—C14—C15	118.8 (8)
C10—P1—Au	113.5 (2)	C13—C14—H14	120.6
C22—P1—Au	112.5 (2)	C15—C14—H14	120.6
C1—O1—C8	116.9 (6)	C10—C15—C14	120.5 (8)
C1—N1—C2	123.2 (7)	C10—C15—H15	119.7
O2—N2—O3	123.5 (7)	C14—C15—H15	119.7
O2—N2—C5	119.3 (8)	C21—C16—C17	119.3 (7)
O3—N2—C5	117.1 (8)	C21—C16—P1	119.2 (6)
N1—C1—O1	120.4 (7)	C17—C16—P1	121.4 (6)
N1—C1—S1	126.9 (6)	C19—C17—C16	119.4 (8)
O1—C1—S1	112.7 (6)	C19—C17—H17	120.3
C7—C2—C3	118.4 (7)	C16—C17—H17	120.3
C7—C2—N1	119.4 (7)	C20—C18—C19	120.8 (8)
C3—C2—N1	122.2 (8)	C20—C18—H18	119.6
C4—C3—C2	120.7 (8)	C19—C18—H18	119.6
C4—C3—H3	119.6	C18—C19—C17	120.0 (8)
C2—C3—H3	119.6	C18—C19—H19	120.0
C3—C4—C5	119.5 (8)	C17—C19—H19	120.0
C3—C4—H4	120.2	C18—C20—C21	119.6 (9)
C5—C4—H4	120.2	C18—C20—H20	120.2
C4—C5—C6	121.9 (7)	C21—C20—H20	120.2
C4—C5—N2	119.2 (8)	C16—C21—C20	120.8 (8)
C6—C5—N2	118.9 (8)	C16—C21—H21	119.6
C7—C6—C5	117.4 (8)	C20—C21—H21	119.6
C7—C6—H6	121.3	C23—C22—C27	120.6 (7)
C5—C6—H6	121.3	C23—C22—P1	117.4 (6)
C2—C7—C6	122.0 (8)	C27—C22—P1	122.0 (6)
C2—C7—H7	119.0	C22—C23—C24	119.7 (8)
C6—C7—H7	119.0	C22—C23—H23	120.2
O1—C8—C9	106.9 (7)	C24—C23—H23	120.2
O1—C8—H8A	110.3	C25—C24—C23	118.7 (8)
C9—C8—H8A	110.3	C25—C24—H24	120.7
O1—C8—H8B	110.3	C23—C24—H24	120.7
C9—C8—H8B	110.3	C26—C25—C24	121.0 (8)
H8A—C8—H8B	108.6	C26—C25—H25	119.5
C8—C9—H9A	109.5	C24—C25—H25	119.5
C8—C9—H9B	109.5	C25—C26—C27	120.1 (8)
H9A—C9—H9B	109.5	C25—C26—H26	119.9
C8—C9—H9C	109.5	C27—C26—H26	119.9
H9A—C9—H9C	109.5	C26—C27—C22	120.0 (8)
H9B—C9—H9C	109.5	C26—C27—H27	120.0
C11—C10—C15	119.9 (7)	C22—C27—H27	120.0
C11—C10—P1	121.6 (5)	Cl2—C28—Cl1	112.4 (6)
C15—C10—P1	118.5 (6)	Cl2—C28—H28A	109.1
C12—C11—C10	120.0 (7)	Cl1—C28—H28A	109.1
C12—C11—H11	120.0	Cl2—C28—H28B	109.1
C10—C11—H11	120.0	Cl1—C28—H28B	109.1
C11—C12—C13	119.7 (8)	H28A—C28—H28B	107.9
			
C2—N1—C1—O1	−174.9 (7)	C12—C13—C14—C15	1.8 (14)
C2—N1—C1—S1	5.0 (13)	C11—C10—C15—C14	−0.6 (12)
C8—O1—C1—N1	1.3 (11)	P1—C10—C15—C14	180.0 (6)
C8—O1—C1—S1	−178.6 (6)	C13—C14—C15—C10	−0.6 (13)
Au—S1—C1—N1	170.3 (8)	C10—P1—C16—C21	−92.8 (7)
Au—S1—C1—O1	−9.8 (6)	C22—P1—C16—C21	154.5 (7)
C1—N1—C2—C7	−122.3 (9)	Au—P1—C16—C21	31.8 (7)
C1—N1—C2—C3	60.6 (12)	C10—P1—C16—C17	85.6 (7)
C7—C2—C3—C4	−1.1 (13)	C22—P1—C16—C17	−27.1 (8)
N1—C2—C3—C4	176.0 (8)	Au—P1—C16—C17	−149.8 (6)
C2—C3—C4—C5	−0.3 (13)	C21—C16—C17—C19	0.7 (13)
C3—C4—C5—C6	1.0 (13)	P1—C16—C17—C19	−177.6 (7)
C3—C4—C5—N2	−178.7 (8)	C20—C18—C19—C17	0.0 (15)
O2—N2—C5—C4	−7.6 (12)	C16—C17—C19—C18	0.3 (14)
O3—N2—C5—C4	171.2 (8)	C19—C18—C20—C21	−1.2 (17)
O2—N2—C5—C6	172.6 (8)	C17—C16—C21—C20	−2.0 (14)
O3—N2—C5—C6	−8.5 (12)	P1—C16—C21—C20	176.4 (8)
C4—C5—C6—C7	−0.4 (14)	C18—C20—C21—C16	2.3 (16)
N2—C5—C6—C7	179.4 (8)	C16—P1—C22—C23	−68.1 (6)
C3—C2—C7—C6	1.8 (13)	C10—P1—C22—C23	179.5 (5)
N1—C2—C7—C6	−175.4 (8)	Au—P1—C22—C23	54.5 (6)
C5—C6—C7—C2	−1.1 (14)	C16—P1—C22—C27	113.7 (6)
C1—O1—C8—C9	178.0 (7)	C10—P1—C22—C27	1.3 (7)
C16—P1—C10—C11	−21.5 (7)	Au—P1—C22—C27	−123.7 (6)
C22—P1—C10—C11	90.1 (6)	C27—C22—C23—C24	−0.6 (11)
Au—P1—C10—C11	−145.5 (5)	P1—C22—C23—C24	−178.9 (6)
C16—P1—C10—C15	157.9 (6)	C22—C23—C24—C25	0.9 (11)
C22—P1—C10—C15	−90.4 (6)	C23—C24—C25—C26	−1.3 (13)
Au—P1—C10—C15	34.0 (6)	C24—C25—C26—C27	1.4 (13)
C15—C10—C11—C12	0.5 (11)	C25—C26—C27—C22	−1.2 (12)
P1—C10—C11—C12	179.9 (6)	C23—C22—C27—C26	0.8 (11)
C10—C11—C12—C13	0.7 (12)	P1—C22—C27—C26	178.9 (6)
C11—C12—C13—C14	−1.9 (14)		

### Hydrogen-bond geometry (Å, °)

**Table:**

*D*—H···*A*	*D*—H	H···*A*	*D*···*A*	*D*—H···*A*
C23—H23···N1^i^	0.94	2.55	3.318 (11)	139
C14—H14···O3^ii^	0.94	2.47	3.366 (12)	160
C28—H28a···O1^iii^	0.98	2.52	3.330 (13)	140
C28—H28b···S1^iv^	0.98	2.86	3.617 (11)	134

Symmetry codes: (i) −*x*, −*y*+1, −*z*; (ii) −*x*+1, −*y*, −*z*; (iii) *x*+1, *y*, *z*+1; (iv) −*x*+1, −*y*, −*z*+1.
